# Supplementary figures and images for: Antiviral efficacy of short-hairpin RNAs and artificial microRNAs targeting foot-and-mouth disease virus
Source: PeerJ. 2021 Jun 9;9:e11227. doi: 10.7717/peerj.11227 (PMC8197037; doi:10.7717/peerj.11227)

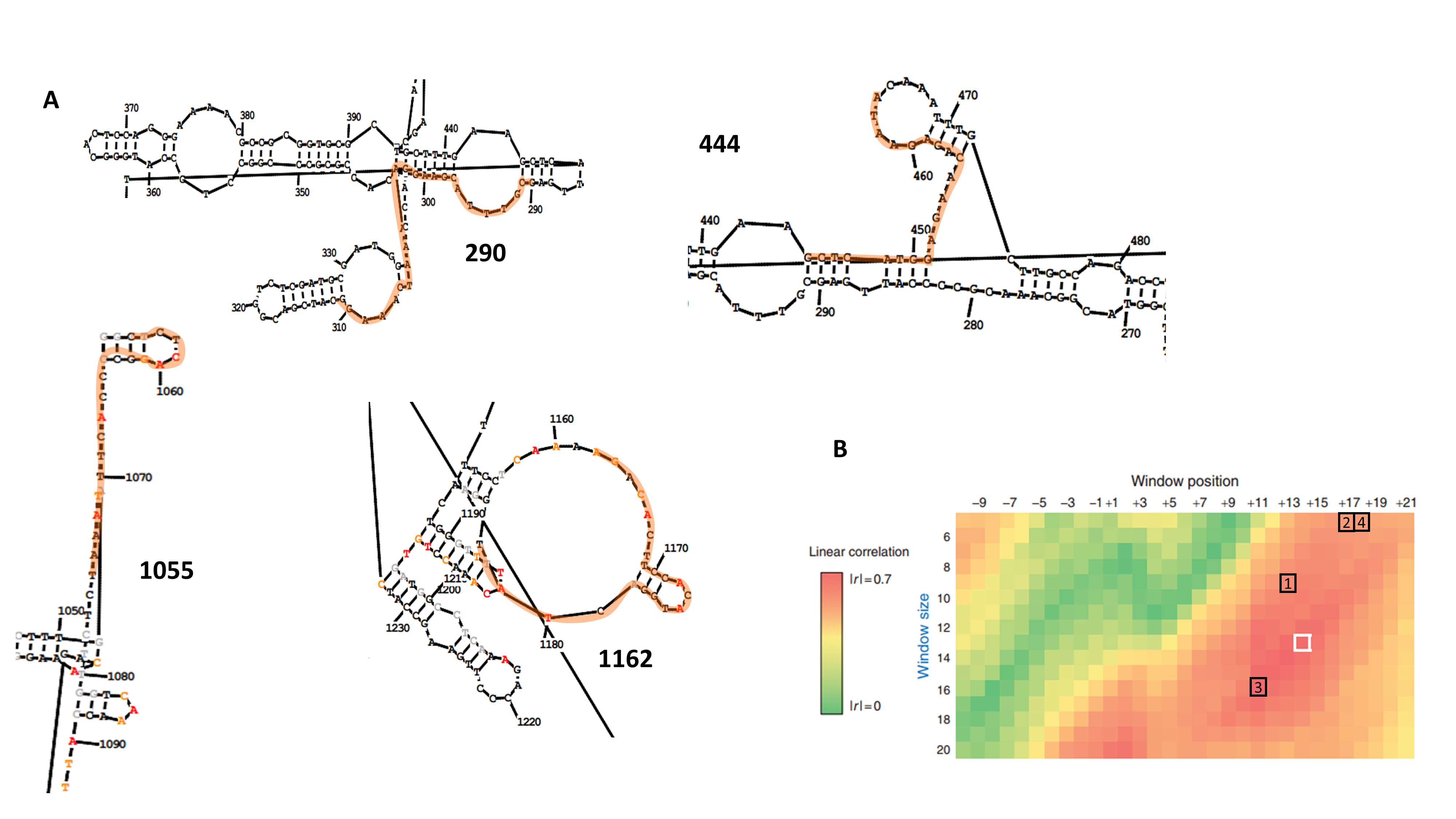

Supplement: Supplemental Information 2 — (A) Local RNA structure in the proximity of selected target sequences. RNA structure was predicted using RNAstructure software and SHAPE data covering a 518-nt region spanning positions 892–1,410 of FMDV 3D (data not shown). Target sequences are indicated in orange. Colored nucleotides in sequences 1,055–1,075 and 1,162–1,182 represent SHAPE reactivity (red: 0.7–1.0; orange: 0.3–0.7; black: 0.0–0.3; grey: no reactivity data). Nucleotides are numbered according to their position in 3Dpol-coding sequence. (B) Correlation between length and starting position of the accessibility window and shRNA efficacy for the training dataset described in Low et al. (Mol Therapy 20:820-828. 2012, modified with permission). The white box indicates the optimal accesible window reported by the authors, starting at position 14 and with a length of 13 nt. Black boxes represent the target sequences selected in this work (1: 290; 2: 444; 3: 1055; 4: 1162). [file peerj-09-11227-s002.png]

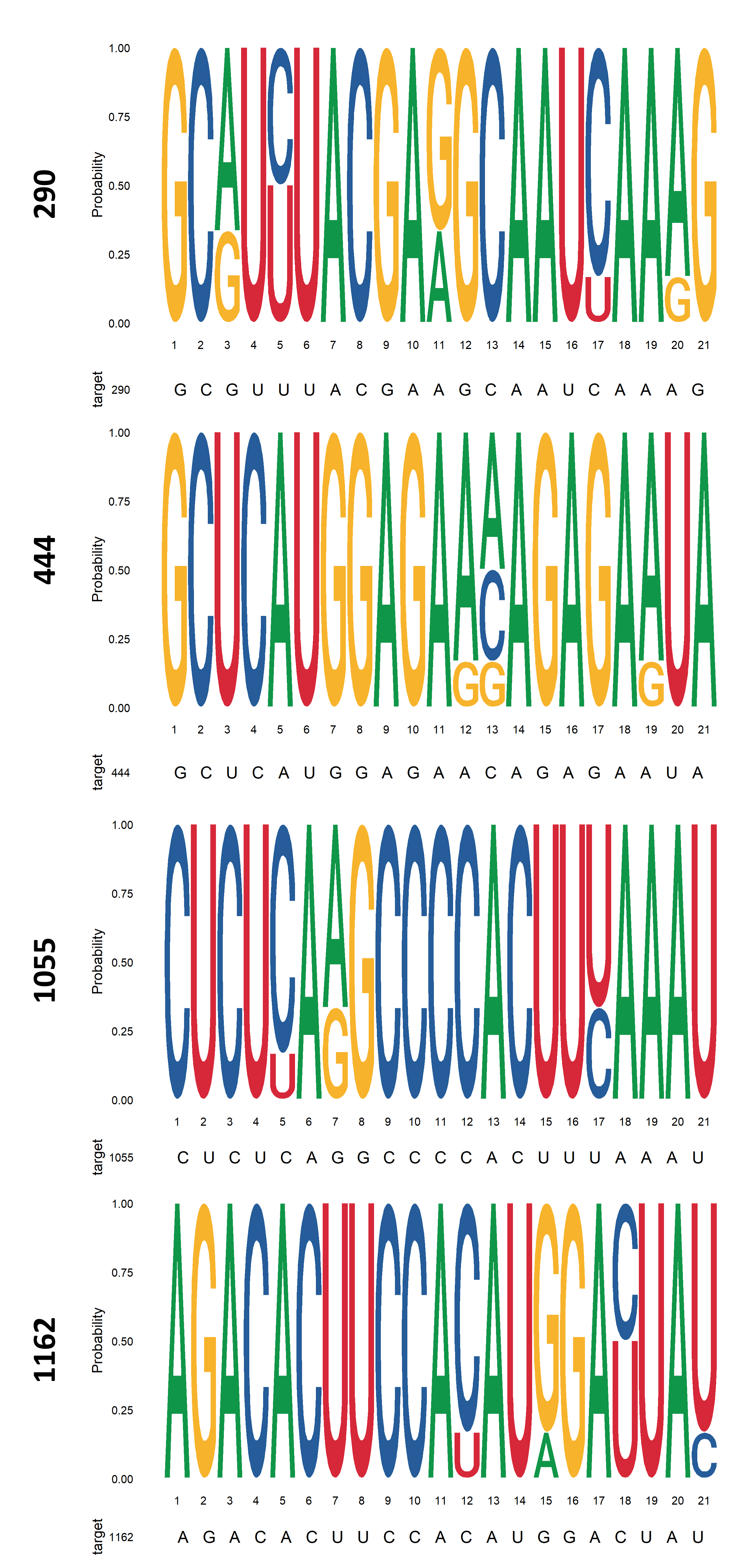

Supplement: Supplemental Information 3 — (C) Conservation of selected target sequences among prototypic FMDV strains of endemic pool 7, which includes serotypes A and O (http://www.foot-and-mouth.org). GenBank accession numbers of prototypic strains are AY593768.1, AY593780.1, AY593815.1, M10975.1. The target sequence derived from FMDV strain A01L used in this study (GenBank accession No. KY404934) is indicated under each logo. [file peerj-09-11227-s003.png]

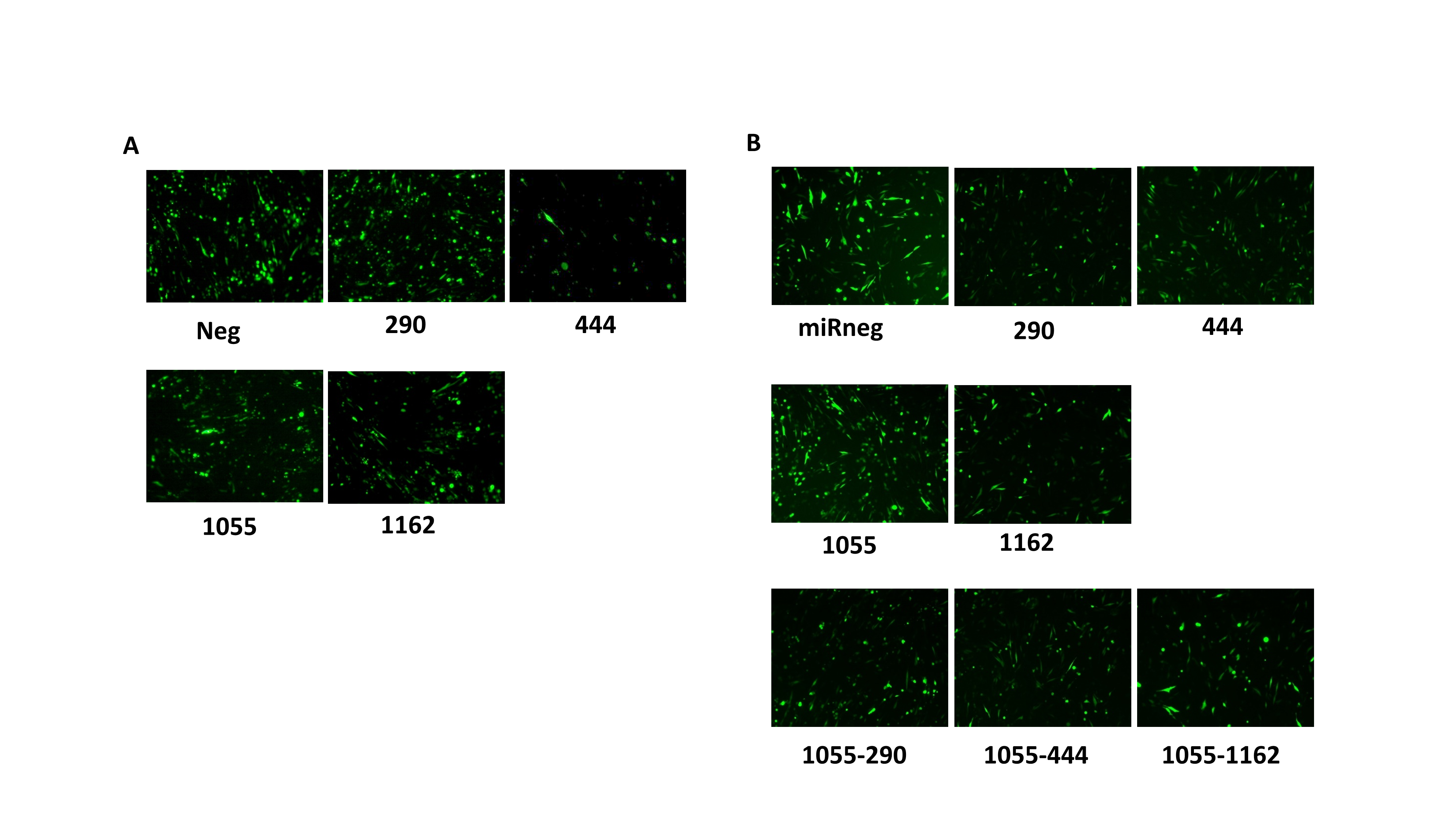

Supplement: Supplemental Information 4 — (A) shRNAFMDV, (B) amiRNAFMDV and dual amiRNAFMDV. Magnification 400X [file peerj-09-11227-s004.png]

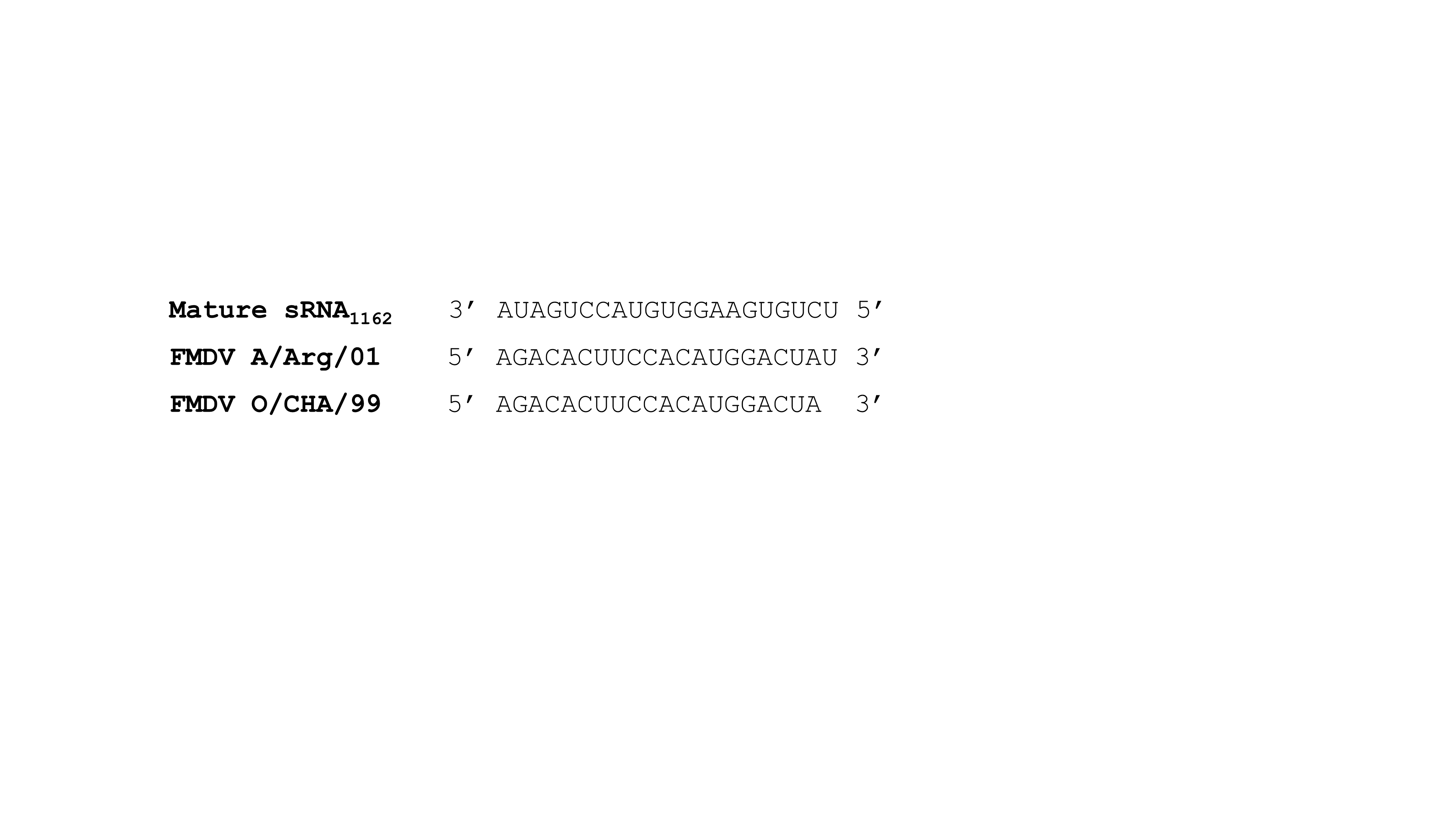

Supplement: Supplemental Information 5 [file peerj-09-11227-s005.png]

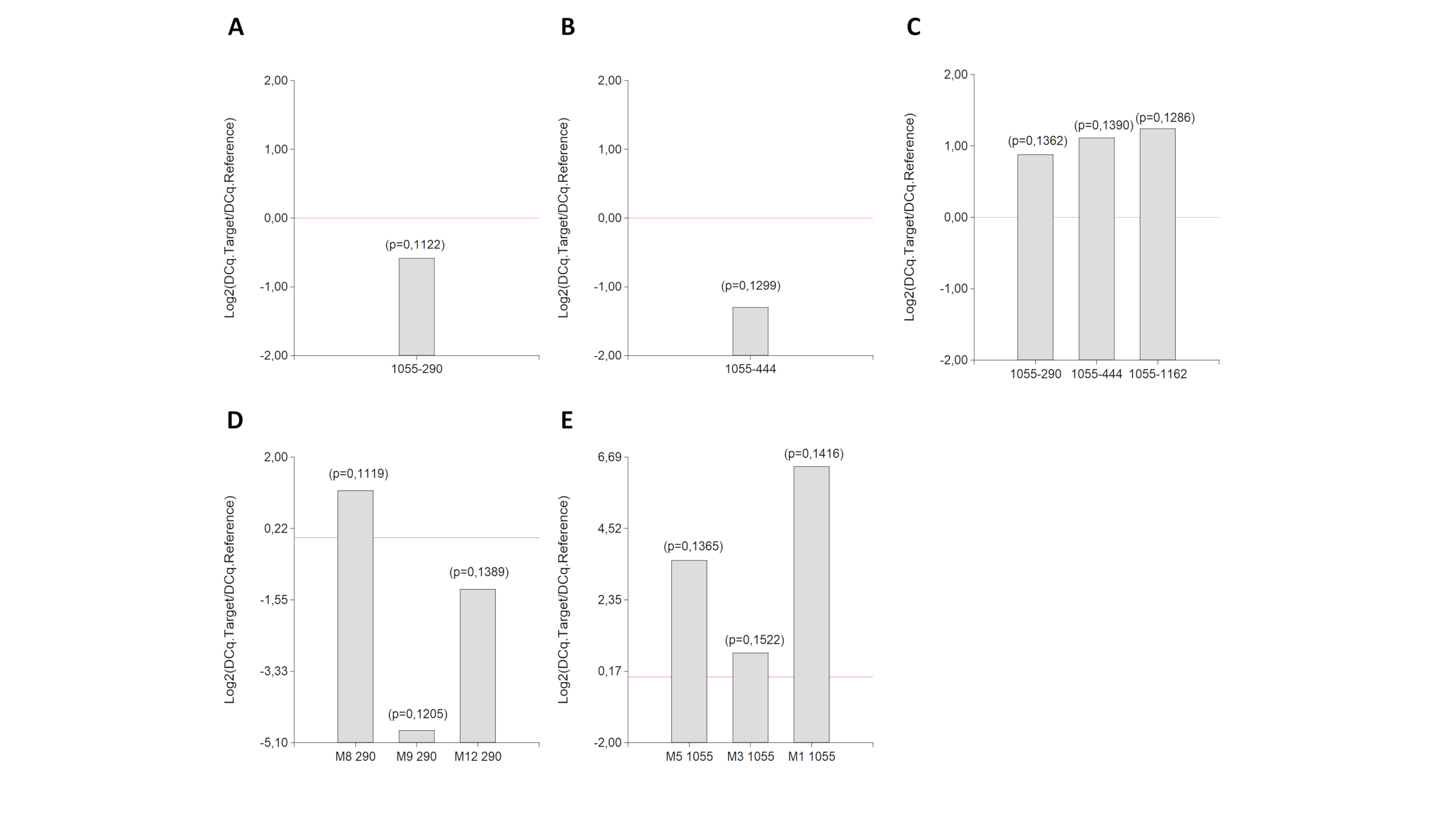

Supplement: Supplemental Information 6 — Total RNA was isolated from BHK-21 cells stably transfected with the corresponding single or dual amiRNA-expressing plasmids, and mature amiRNAs were quantitated by RT-stem loop real time PCR as described by Chen et al (Chen, 2005). Mature amiRNA290 (A), amiRNA444 (B) and amiRNA_1055 ©expression from polycistronic vectors was normalized by glyceraldehyde-3-phosphate deshydrogenase (GAPDH, A, B) or endogenous miRNA-706 ©and relative expression to the same amiRNA in mono-amiRNA polyclonal cells (horizontal line) was calculated by the 2−ΔΔ CT method. (D, E) Mature amiRNAs were quantitated in clonal cell lines and relative expression to amiRNA290 (D) or amiRNA1055 (E) (horizontal lines) was calculated as in (A). [file peerj-09-11227-s006.png]

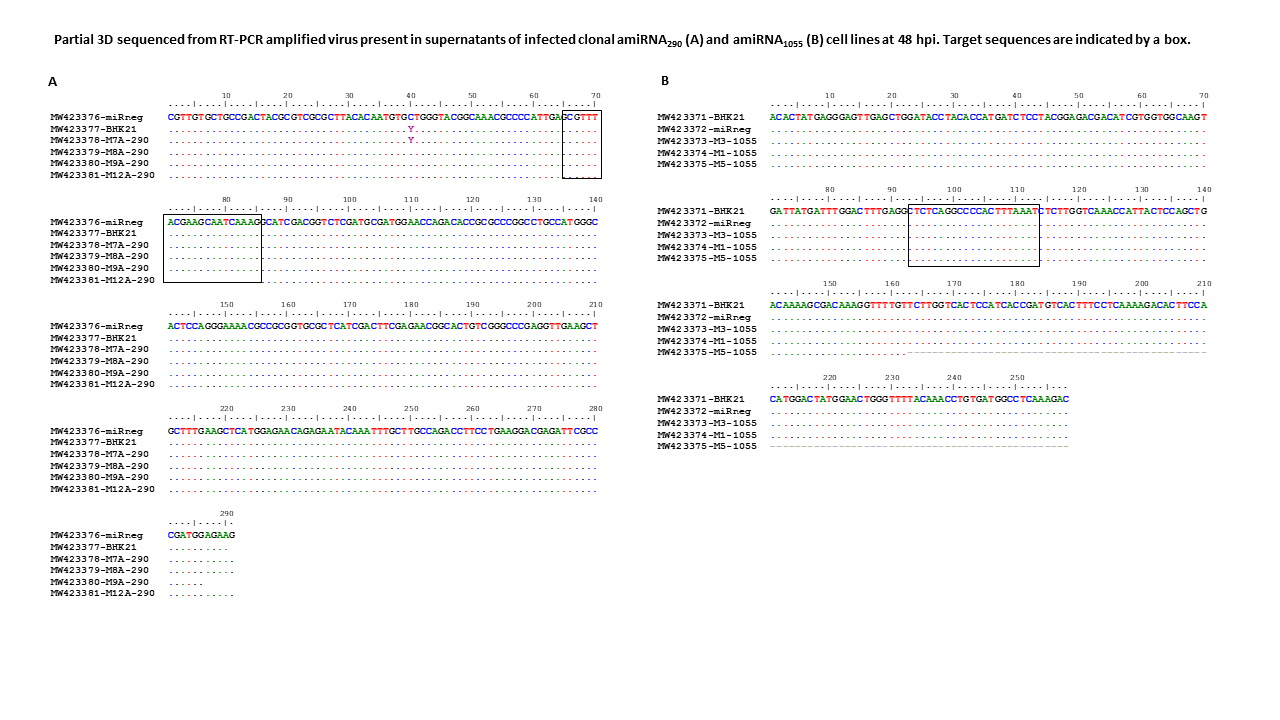

Supplement: Supplemental Information 7 — Target sequences are indicated by a box. [file peerj-09-11227-s007.png]
