# Supplementary material for: Antiviral efficacy of short-hairpin RNAs and artificial microRNAs targeting foot-and-mouth disease virus
Source: PeerJ. 2021 Jun 9;9:e11227. doi: 10.7717/peerj.11227 (PMC8197037; doi:10.7717/peerj.11227)
Supplement: Supplemental Information 8 [file peerj-09-11227-s008.docx]

| **Name** | **Sequence (5’-3’)^a^** | **Application** |
| --- | --- | --- |
| **290top** | TGCTGCTTTGATTGCTTCGTAAACGCGTTTTGGCCACTGACTGACGCGTTTACAGCAATCAAAG | Cloning of pre-amiR_290_ |
| **290bot** | CCTGCTTTGATTGCTGTAAACGCGTCAGTCAGTGGCCAAAACGCGTTTACGAAGCAATCAAAGC |  |
|  |  |  |
| **444top** | TGCTGTATTCTCTGTTCTCCATGAGCGTTTTGGCCACTGACTGACGCTCATGGAACAGAGAATA | Cloning of pre-amiR_444_ |
| **444bot** | CCTGTATTCTCTGTTCCATGAGCGTCAGTCAGTGGCCAAAACGCTCATGGAGAACAGAGAATAC |  |
|  |  |  |
| **1055top** | TGCTGATTTAAAGTGGGGCCTGAGAGGTTTTGGCCACTGACTGACCTCTCAGGCCACTTTAAAT | Cloning of pre-amiR_1055_ |
| **1055bot** | CCTGATTTAAAGTGGCCTGAGAGGTCAGTCAGTGGCCAAAACCTCTCAGGCCCCACTTTAAATC |  |
|  |  |  |
| **1162top** | TGCTGATAGTCCATGTGGAAGTGTCTGTTTTGGCCACTGACTGACAGACACTTACATGGACTAT | Cloning of pre-amiR_1162_ |
| **1162bot** | CCTGATAGTCCATGTAAGTGTCTGTCAGTCAGTGGCCAAAACAGACACTTCCACATGGACTATC |  |
|  |  |  |
| **U6for SpeI** | AACTAGTGAGGGCCTATTTCCCATG | Amplification of U6 promoter |
| **U6rev XhoI** | ACTCGAGAAACAGGTCTTCTCGAAG |  |
|  |  |  |
| **sh290for** | CACCGCTTTGATTGCTTCGTAAACGCTTCAAGAGAGCGTTTACGAAGCAATCAAAGCTTTTTT | Cloning of psh_290_ |
| **sh290rev** | AAACAAAAAAGCTTTGATTGCTTCGTAAACGCTCTCTTGAAGCGTTTACGAAGCAATCAAAGC |  |
|  |  |  |
| **sh444for** | CACCGTATTCTCTGTTCTCCATGAGCTTCAAGAGAGCTCATGGAGAACAGAGAATACTTTTTT | Cloning of psh_444_ |
| **sh444rev** | AAACAAAAAAGTATTCTCTGTTCTCCATGAGCTCTCTTGAAGCTCATGGAGAACAGAGAATAC |  |
|  |  |  |
| **sh1055for** | CACCGATTTAAAGTGGGGCCTGAGAGTTCAAGAGACTCTCAGGCCCCACTTTAAATCTTTTTT | Cloning of psh_1055_ |
| **sh1055rev** | AAACAAAAAAGATTTAAAGTGGGGCCTGAGAGTCTCTTGAACTCTCAGGCCCCACTTTAAATC |  |
|  |  |  |
| **sh1162for** | CACCGATAGTCCATGTGGAAGTGTCTTTCAAGAGAAGACACTTCCACATGGACTATCTTTTTT | Cloning of psh_1162_ |
| **sh1162rev** | AAACAAAAAAGATAGTCCATGTGGAAGTGTCTTCTCTTGAAAGACACTTCCACATGGACTATC |  |
|  |  |  |
| **Shnegfor** | CACCGAAATGTACTGCGCGTGGAGACTTCAAGAGAGTCTCCACGCGCAGTACATTTCTTTTTT | Cloning of psh_neg_ |
| **Shnegfor** | AAACAAAAAAGAAATGTACTGCGCGTGGAGACTCTCTTGAAGTCTCCACGCGCAGTACATTTC |  |
|  |  |  |
| **EGFPfor KpnI** | GGTACCATGGTGAGCAAGGGCG | Construction of pEGFP.3D |
| **EGFPrev EcoRI** | GAATTCTTACTTGTACAGCTCGTCC |  |
|  |  |  |
| **3Dfor EcoRI** | GGTACCATGGTGAGCAAGGGCG | Construction of pEGFP.3D |
| **3Drev EcoRI** | GAATTCTTACTTGTACAGCTCGTCC |  |
|  |  |  |
| **miRNAseq for** | TCCCAAGCTGGCTAGTTAAG | Evaluation of transgenic cell lines |
| **miRNAseq rev** | CTCTAGATCAACCACTTTGT |  |

^a^ Restriction sites used for cloning are underlined
