# Supplementary material for: Antiviral efficacy of short-hairpin RNAs and artificial microRNAs targeting foot-and-mouth disease virus
Source: PeerJ. 2021 Jun 9;9:e11227. doi: 10.7717/peerj.11227 (PMC8197037; doi:10.7717/peerj.11227)
Supplement: Supplemental Information 9 [file peerj-09-11227-s009.docx]

| **WORST RANK** | **Position*^a^*** | **Target sequence (5’-3’)** | **siRNA sequence (5’-3’)** | **Access**  **8 nts*^b^*** | **Access**  **16 nts*^c^*** | **Energy A.*^d^*** | **Sequence A.*^e^*** | **Self Folding*^f^*** | **Free End*^g^*** |
| --- | --- | --- | --- | --- | --- | --- | --- | --- | --- |
| 37 | 468 | GGAGAACAGAGAAUACAAA | UUUGUAUUCUCUGUUCUCC | 0.6886 | 0.0269 | 0.8707 | 1.0000 | 1.0000 | 10.000 |
| 37 | 309 | CGUUUACGAAGCAAUCAAA | UUUGAUUGCUUCGUAAACG | 0.2148 | 0.0374 | 0.7759 | 1.0000 | 1.0000 | 10.000 |
| 39 | 308 | GCGUUUACGAAGCAAUCAA | UUGAUUGCUUCGUAAACGC | 0.2121 | 0.0266 | 0.7759 | 1.0000 | 1.0000 | 10.000 |
| 43 | 1038 | CGUGGUGGCAAGUGAUUAU | AUAAUCACUUGCCACCACG | 0.2700 | 0.0246 | 0.7328 | 1.0000 | 1.0000 | 10.000 |
| 48 | 1075 | CUCAGGCCCCACUUUAAAU | AUUUAAAGUGGGGCCUGAG | 0.3822 | 0.0226 | 0.8017 | 0.7500 | 1.0000 | 10.000 |
| 53 | 467 | UGGAGAACAGAGAAUACAA | UUGUAUUCUCUGUUCUCCA | 0.6112 | 0.0210 | 0.7069 | 0.7500 | 1.0000 | 10.000 |
| 54 | 1080 | GCCCCACUUUAAAUCUCUU | AAGAGAUUUAAAGUGGGGC | 0.1590 | 0.0969 | 0.8621 | 1.0000 | 1.0000 | 10.000 |
| 56 | 597 | GAUGAUGAUAGGCAGAUUU | AAAUCUGCCUAUCAUCAUC | 0.1548 | 0.0480 | 0.6810 | 0.7500 | 1.0000 | 10.000 |
| 58 | 1073 | CUCUCAGGCCCCACUUUAA | UUAAAGUGGGGCCUGAGAG | 0.2421 | 0.0178 | 0.7759 | 0.7500 | 1.0000 | 10.000 |
| 60 | 1161 | CGAUGUCACUUUCCUCAAA | UUUGAGGAAAGUGACAUCG | 0.3612 | 0.0175 | 0.7931 | 1.0000 | 1.0000 | 10.000 |
| 60 | 469 | GAGAACAGAGAAUACAAAU | AUUUGUAUUCUCUGUUCUC | 0.1469 | 0.0213 | 0.8017 | 0.7500 | 1.0000 | 10.000 |
| 61 | 595 | AGGAUGAUGAUAGGCAGAU | AUCUGCCUAUCAUCAUCCU | 0.1508 | 0.0383 | 0.6638 | 0.7500 | 1.0000 | 10.000 |
| 62 | 596 | GGAUGAUGAUAGGCAGAUU | AAUCUGCCUAUCAUCAUCC | 0.1441 | 0.0420 | 0.8621 | 1.0000 | 1.0000 | 10.000 |
| 66 | 1072 | GCUCUCAGGCCCCACUUUA | UAAAGUGGGGCCUGAGAGC | 0.2242 | 0.0155 | 0.8190 | 1.0000 | 1.0000 | 10.000 |
| 66 | 470 | AGAACAGAGAAUACAAAUU | AAUUUGUAUUCUCUGUUCU | 0.1279 | 0.0204 | 0.7155 | 0.7500 | 1.0000 | 10.000 |
| 67 | 1160 | CCGAUGUCACUUUCCUCAA | UUGAGGAAAGUGACAUCGG | 0.3815 | 0.0154 | 0.7672 | 1.0000 | 1.0000 | 10.000 |
| 72 | 471 | GAACAGAGAAUACAAAUUU | AAAUUUGUAUUCUCUGUUC | 0.1215 | 0.0227 | 0.6724 | 0.7500 | 1.0000 | 10.000 |
| 74 | 992 | GAGUUGAGCUGGAUACCUA | UAGGUAUCCAGCUCAACUC | 0.3270 | 0.0527 | 0.6379 | 0.7500 | 1.0000 | 10.000 |
| 77 | 1246 | GCCAUCCUCUCCUUUGCAC | GUGCAAAGGAGAGGAUGGC | 0.3229 | 0.1168 | 0.7069 | 0.7500 | 1.0000 | 10.000 |
| 77 | 1037 | UCGUGGUGGCAAGUGAUUA | UAAUCACUUGCCACCACGA | 0.2855 | 0.0125 | 0.7586 | 0.7500 | 1.0000 | 10.000 |
| 78 | 1074 | UCUCAGGCCCCACUUUAAA | UUUAAAGUGGGGCCUGAGA | 0.3020 | 0.0211 | 0.7241 | 0.7500 | 1.0000 | 10.000 |
| 79 | 1079 | GGCCCCACUUUAAAUCUCU | AGAGAUUUAAAGUGGGGCC | 0.3753 | 0.0808 | 0.7759 | 0.7500 | 1.0000 | 10.000 |
| 81 | 1369 | GAGAUACCAAGCUACAGAU | AUCUGUAGCUUGGUAUCUC | 0.4263 | 0.0117 | 0.6293 | 0.7500 | 1.0000 | 10.000 |
| 84 | 1162 | GAUGUCACUUUCCUCAAAA | UUUUGAGGAAAGUGACAUC | 0.3533 | 0.0178 | 0.6810 | 0.7500 | 1.0000 | 10.000 |
| 87 | 922 | GCGACAAGCAUCAUCAACA | UGUUGAUGAUGCUUGUCGC | 0.0886 | 0.0102 | 0.7069 | 0.7500 | 0.9674 | 0.7500 |
| 89 | 1042 | GUGGCAAGUGAUUAUGAUU | AAUCAUAAUCACUUGCCAC | 0.0837 | 0.0696 | 0.7845 | 0.7500 | 1.0000 | 10.000 |
| 94 | 921 | CGCGACAAGCAUCAUCAAC | GUUGAUGAUGCUUGUCGCG | 0.1078 | 0.0116 | 0.7759 | 0.7500 | 0.9511 | 0.7500 |
| 95 | 1043 | UGGCAAGUGAUUAUGAUUU | AAAUCAUAAUCACUUGCCA | 0.0654 | 0.0494 | 0.8534 | 0.7500 | 1.0000 | 10.000 |
| 98 | 935 | UCAACACGAUUUUGAACAA | UUGUUCAAAAUCGUGUUGA | 0.1693 | 0.0513 | 0.5862 | 0.7500 | 1.0000 | 10.000 |
| 98 | 570 | GCCUGUUGAACACAUUCUU | AAGAAUGUGUUCAACAGGC | 0.0637 | 0.0286 | 0.8621 | 1.0000 | 0.9565 | 0.6250 |
| 99 | 475 | AGAGAAUACAAAUUUGCUU | AAGCAAAUUUGUAUUCUCU | 0.0849 | 0.0312 | 0.5862 | 0.7500 | 1.0000 | 10.000 |
| 102 | 1153 | UCCAUCACCGAUGUCACUU | AAGUGACAUCGGUGAUGGA | 0.0614 | 0.0112 | 0.7328 | 0.7500 | 0.9076 | 0.7500 |
| 103 | 464 | UCAUGGAGAACAGAGAAUA | UAUUCUCUGUUCUCCAUGA | 0.0621 | 0.0064 | 0.6897 | 0.7500 | 1.0000 | 10.000 |
| 105 | 672 | CCCUGAUGUUGAUUGGCAA | UUGCCAAUCAACAUCAGGG | 0.0962 | 0.0062 | 0.8448 | 1.0000 | 1.0000 | 10.000 |
| 105 | 571 | CCUGUUGAACACAUUCUUU | AAAGAAUGUGUUCAACAGG | 0.0587 | 0.0209 | 0.8448 | 1.0000 | 0.9620 | 0.7500 |
| 106 | 593 | CCAGGAUGAUGAUAGGCAG | CUGCCUAUCAUCAUCCUGG | 0.1620 | 0.0355 | 0.5690 | 0.7500 | 1.0000 | 10.000 |
| 107 | 1251 | CCUCUCCUUUGCACGCCGU | ACGGCGUGCAAAGGAGAGG | 0.0601 | 0.0231 | 0.5690 | 0.7500 | 1.0000 | 10.000 |
| 108 | 673 | CCUGAUGUUGAUUGGCAAA | UUUGCCAAUCAACAUCAGG | 0.1147 | 0.0058 | 0.8448 | 1.0000 | 1.0000 | 10.000 |
| 109 | 101 | GUGUGUUCAACCCUGAGUU | AACUCAGGGUUGAACACAC | 0.0570 | 0.0058 | 0.6466 | 0.7500 | 1.0000 | 10.000 |
| 111 | 938 | ACACGAUUUUGAACAACAU | AUGUUGUUCAAAAUCGUGU | 0.2937 | 0.0485 | 0.5603 | 0.7500 | 1.0000 | 10.000 |
| 112 | 869 | CCUAUGAGAACAAACGCAU | AUGCGUUUGUUCUCAUAGG | 0.0961 | 0.0056 | 0.6897 | 1.0000 | 1.0000 | 10.000 |
| 113 | 572 | CUGUUGAACACAUUCUUUA | UAAAGAAUGUGUUCAACAG | 0.0530 | 0.0165 | 0.7500 | 0.7500 | 0.9620 | 0.6250 |
| 113 | 1159 | ACCGAUGUCACUUUCCUCA | UGAGGAAAGUGACAUCGGU | 0.3913 | 0.0146 | 0.6207 | 0.5000 | 1.0000 | 10.000 |
| 113 | 870 | CUAUGAGAACAAACGCAUU | AAUGCGUUUGUUCUCAUAG | 0.0809 | 0.0055 | 0.6638 | 0.7500 | 1.0000 | 10.000 |
| 114 | 523 | CCGAUGGAGAAAGUACGUG | CACGUACUUUCUCCAUCGG | 0.1407 | 0.0055 | 0.6207 | 0.7500 | 1.0000 | 10.000 |
| 115 | 1173 | CCUCAAAAGACACUUCCAC | GUGGAAGUGUCUUUUGAGG | 0.0536 | 0.0237 | 0.5517 | 0.7500 | 1.0000 | 10.000 |
| 116 | 1154 | CCAUCACCGAUGUCACUUU | AAAGUGACAUCGGUGAUGG | 0.0502 | 0.0132 | 0.8534 | 1.0000 | 0.9076 | 0.6250 |
| 117 | 920 | CCGCGACAAGCAUCAUCAA | UUGAUGAUGCUUGUCGCGG | 0.2314 | 0.0053 | 0.7672 | 1.0000 | 0.9348 | 0.6250 |
| 118 | 1364 | UCUUUGAGAUACCAAGCUA | UAGCUUGGUAUCUCAAAGA | 0.1002 | 0.0076 | 0.5517 | 0.7500 | 1.0000 | 10.000 |
| 118 | 1135 | GGUUUUGUUCUUGGUCACU | AGUGACCAAGAACAAAACC | 0.0643 | 0.0051 | 0.6466 | 0.7500 | 1.0000 | 10.000 |
| 118 | 306 | GAGCGUUUACGAAGCAAUC | GAUUGCUUCGUAAACGCUC | 0.2048 | 0.0057 | 0.6293 | 0.5000 | 1.0000 | 10.000 |
| 119 | 100 | GGUGUGUUCAACCCUGAGU | ACUCAGGGUUGAACACACC | 0.1242 | 0.0050 | 0.6466 | 0.7500 | 1.0000 | 10.000 |
| 120 | 1077 | CAGGCCCCACUUUAAAUCU | AGAUUUAAAGUGGGGCCUG | 0.3600 | 0.0380 | 0.5603 | 0.5000 | 1.0000 | 10.000 |
| 120 | 1036 | AUCGUGGUGGCAAGUGAUU | AAUCACUUGCCACCACGAU | 0.2778 | 0.0049 | 0.6724 | 0.5000 | 1.0000 | 10.000 |
| 121 | 168 | UGUCCUCGAUGAAGUCAUU | AAUGACUUCAUCGAGGACA | 0.0774 | 0.0049 | 0.7069 | 0.7500 | 0.9457 | 0.8750 |
| 121 | 1076 | UCAGGCCCCACUUUAAAUC | GAUUUAAAGUGGGGCCUGA | 0.3783 | 0.0255 | 0.5517 | 0.5000 | 1.0000 | 10.000 |
| 122 | 988 | GAGGGAGUUGAGCUGGAUA | UAUCCAGCUCAACUCCCUC | 0.1280 | 0.0049 | 0.7672 | 0.7500 | 1.0000 | 10.000 |
| 122 | 1174 | CUCAAAAGACACUUCCACA | UGUGGAAGUGUCUUUUGAG | 0.0567 | 0.0197 | 0.5948 | 0.5000 | 1.0000 | 10.000 |
| 123 | 1078 | AGGCCCCACUUUAAAUCUC | GAGAUUUAAAGUGGGGCCU | 0.3696 | 0.0520 | 0.5345 | 0.5000 | 1.0000 | 10.000 |
| 124 | 476 | GAGAAUACAAAUUUGCUUG | CAAGCAAAUUUGUAUUCUC | 0.0893 | 0.0488 | 0.6638 | 0.5000 | 1.0000 | 10.000 |
| 124 | 573 | UGUUGAACACAUUCUUUAC | GUAAAGAAUGUGUUCAACA | 0.0498 | 0.0283 | 0.5345 | 0.5000 | 0.9891 | 10.000 |
| 124 | 169 | GUCCUCGAUGAAGUCAUUU | AAAUGACUUCAUCGAGGAC | 0.0632 | 0.0046 | 0.8190 | 0.7500 | 0.9457 | 0.7500 |
| 125 | 836 | GGAUUCUAAAGACUCUCGU | ACGAGAGUCUUUAGAAUCC | 0.0549 | 0.0046 | 0.5948 | 0.7500 | 0.9783 | 0.7500 |
| 125 | 1175 | UCAAAAGACACUUCCACAU | AUGUGGAAGUGUCUUUUGA | 0.0463 | 0.0155 | 0.5776 | 0.7500 | 1.0000 | 10.000 |
| 127 | 1044 | GGCAAGUGAUUAUGAUUUG | CAAAUCAUAAUCACUUGCC | 0.0457 | 0.0279 | 0.8534 | 0.7500 | 1.0000 | 10.000 |
| 127 | 745 | GCCUUCGAUGCUAACCACU | AGUGGUUAGCAUCGAAGGC | 0.5197 | 0.0044 | 0.7931 | 0.7500 | 1.0000 | 10.000 |
| 128 | 630 | CUCAAACAACGGACCGCAG | CUGCGGUCCGUUGUUUGAG | 0.0502 | 0.0314 | 0.5259 | 0.5000 | 1.0000 | 10.000 |
| 129 | 60 | CGUGAUGCGCAAAACCAAG | CUUGGUUUUGCGCAUCACG | 0.1164 | 0.0044 | 0.6379 | 0.7500 | 1.0000 | 10.000 |
| 129 | 991 | GGAGUUGAGCUGGAUACCU | AGGUAUCCAGCUCAACUCC | 0.2049 | 0.0391 | 0.5259 | 0.7500 | 1.0000 | 10.000 |
| 130 | 1099 | GGUCAAACCAUUACUCCAG | CUGGAGUAAUGGUUUGACC | 0.1740 | 0.0042 | 0.5690 | 0.7500 | 1.0000 | 10.000 |
| 133 | 183 | CAUUUUCUCCAAACACAAA | UUUGUGUUUGGAGAAAAUG | 0.7079 | 0.0035 | 0.6552 | 0.7500 | 1.0000 | 10.000 |
| 134 | 1147 | GGUCACUCCAUCACCGAUG | CAUCGGUGAUGGAGUGACC | 0.0397 | 0.0089 | 0.6983 | 0.7500 | 0.9239 | 0.6250 |
| 135 | 522 | CCCGAUGGAGAAAGUACGU | ACGUACUUUCUCCAUCGGG | 0.1578 | 0.0034 | 0.7155 | 0.7500 | 1.0000 | 10.000 |
| 135 | 1148 | GUCACUCCAUCACCGAUGU | ACAUCGGUGAUGGAGUGAC | 0.0389 | 0.0124 | 0.6034 | 0.5000 | 0.9076 | 0.6250 |
| 135 | 1368 | UGAGAUACCAAGCUACAGA | UCUGUAGCUUGGUAUCUCA | 0.5130 | 0.0077 | 0.5086 | 0.5000 | 1.0000 | 10.000 |
| 136 | 598 | AUGAUGAUAGGCAGAUUUU | AAAAUCUGCCUAUCAUCAU | 0.1712 | 0.0701 | 0.6552 | 0.5000 | 1.0000 | 10.000 |
| 136 | 709 | GCCCAAUAUAGAAACGUGU | ACACGUUUCUAUAUUGGGC | 0.0387 | 0.0138 | 0.7845 | 0.7500 | 1.0000 | 10.000 |
| 136 | 310 | GUUUACGAAGCAAUCAAAG | CUUUGAUUGCUUCGUAAAC | 0.4382 | 0.0452 | 0.5086 | 0.5000 | 1.0000 | 10.000 |
| 136 | 59 | ACGUGAUGCGCAAAACCAA | UUGGUUUUGCGCAUCACGU | 0.3586 | 0.0033 | 0.6293 | 0.7500 | 0.9946 | 0.6250 |
| 137 | 103 | GUGUUCAACCCUGAGUUUG | CAAACUCAGGGUUGAACAC | 0.0385 | 0.0038 | 0.6466 | 0.5000 | 1.0000 | 10.000 |
| 137 | 631 | UCAAACAACGGACCGCAGA | UCUGCGGUCCGUUGUUUGA | 0.0413 | 0.0267 | 0.5086 | 0.5000 | 1.0000 | 10.000 |
| 137 | 305 | UGAGCGUUUACGAAGCAAU | AUUGCUUCGUAAACGCUCA | 0.1441 | 0.0033 | 0.7241 | 0.7500 | 1.0000 | 10.000 |
| 138 | 99 | CGGUGUGUUCAACCCUGAG | CUCAGGGUUGAACACACCG | 0.0673 | 0.0033 | 0.6034 | 0.7500 | 0.9239 | 0.6250 |
| 138 | 1108 | AUUACUCCAGCUGACAAAA | UUUUGUCAGCUGGAGUAAU | 0.0442 | 0.0148 | 0.5086 | 0.5000 | 1.0000 | 10.000 |
| 139 | 102 | UGUGUUCAACCCUGAGUUU | AAACUCAGGGUUGAACACA | 0.0474 | 0.0033 | 0.7155 | 0.7500 | 1.0000 | 10.000 |
| 140 | 840 | UCUAAAGACUCUCGUGAAC | GUUCACGAGAGUCUUUAGA | 0.0373 | 0.0100 | 0.5776 | 0.5000 | 0.9620 | 10.000 |
| 141 | 1105 | ACCAUUACUCCAGCUGACA | UGUCAGCUGGAGUAAUGGU | 0.0362 | 0.0113 | 0.6379 | 0.5000 | 1.0000 | 10.000 |
| 142 | 182 | UCAUUUUCUCCAAACACAA | UUGUGUUUGGAGAAAAUGA | 0.6971 | 0.0032 | 0.5862 | 0.7500 | 1.0000 | 10.000 |
| 143 | 704 | AUUUCGCCCAAUAUAGAAA | UUUCUAUAUUGGGCGAAAU | 0.2040 | 0.0032 | 0.5086 | 0.5000 | 1.0000 | 10.000 |
| 143 | 839 | UUCUAAAGACUCUCGUGAA | UUCACGAGAGUCUUUAGAA | 0.0379 | 0.0084 | 0.5000 | 0.5000 | 0.9620 | 0.8750 |
| 144 | 1166 | UCACUUUCCUCAAAAGACA | UGUCUUUUGAGGAAAGUGA | 0.1229 | 0.0874 | 0.5172 | 0.5000 | 1.0000 | 10.000 |
| 144 | 304 | UUGAGCGUUUACGAAGCAA | UUGCUUCGUAAACGCUCAA | 0.1477 | 0.0034 | 0.5000 | 0.5000 | 1.0000 | 10.000 |
| 144 | 581 | ACAUUCUUUACACCAGGAU | AUCCUGGUGUAAAGAAUGU | 0.0361 | 0.0031 | 0.5345 | 0.7500 | 1.0000 | 10.000 |
| 145 | 23 | UGAUUGUUGACACCAGAGA | UCUCUGGUGUCAACAAUCA | 0.0409 | 0.0097 | 0.5086 | 0.5000 | 0.9946 | 0.6250 |
| 145 | 936 | CAACACGAUUUUGAACAAC | GUUGUUCAAAAUCGUGUUG | 0.1608 | 0.0486 | 0.5000 | 0.5000 | 1.0000 | 10.000 |
| 146 | 1041 | GGUGGCAAGUGAUUAUGAU | AUCAUAAUCACUUGCCACC | 0.0948 | 0.0031 | 0.6724 | 1.0000 | 1.0000 | 10.000 |
| 146 | 989 | AGGGAGUUGAGCUGGAUAC | GUAUCCAGCUCAACUCCCU | 0.1161 | 0.0131 | 0.6638 | 0.5000 | 1.0000 | 10.000 |
| 147 | 1106 | CCAUUACUCCAGCUGACAA | UUGUCAGCUGGAGUAAUGG | 0.0306 | 0.0298 | 0.7069 | 1.0000 | 1.0000 | 10.000 |
| 147 | 987 | UGAGGGAGUUGAGCUGGAU | AUCCAGCUCAACUCCCUCA | 0.1210 | 0.0030 | 0.5517 | 0.7500 | 1.0000 | 10.000 |
| 147 | 307 | AGCGUUUACGAAGCAAUCA | UGAUUGCUUCGUAAACGCU | 0.2092 | 0.0158 | 0.6207 | 0.5000 | 1.0000 | 10.000 |
| 147 | 1168 | ACUUUCCUCAAAAGACACU | AGUGUCUUUUGAGGAAAGU | 0.1018 | 0.0708 | 0.5000 | 0.5000 | 1.0000 | 10.000 |
| 148 | 594 | CAGGAUGAUGAUAGGCAGA | UCUGCCUAUCAUCAUCCUG | 0.1790 | 0.0421 | 0.5603 | 0.5000 | 1.0000 | 10.000 |
| 148 | 1048 | AGUGAUUAUGAUUUGGACU | AGUCCAAAUCAUAAUCACU | 0.0318 | 0.0212 | 0.5000 | 0.5000 | 1.0000 | 10.000 |
| 149 | 1049 | GUGAUUAUGAUUUGGACUU | AAGUCCAAAUCAUAAUCAC | 0.0301 | 0.0192 | 0.6552 | 0.7500 | 1.0000 | 10.000 |
| 149 | 24 | GAUUGUUGACACCAGAGAU | AUCUCUGGUGUCAACAAUC | 0.0475 | 0.0083 | 0.5000 | 0.7500 | 1.0000 | 10.000 |
| 149 | 181 | GUCAUUUUCUCCAAACACA | UGUGUUUGGAGAAAAUGAC | 0.0916 | 0.0031 | 0.6034 | 0.5000 | 1.0000 | 10.000 |
| 150 | 1361 | GCCUCUUUGAGAUACCAAG | CUUGGUAUCUCAAAGAGGC | 0.0508 | 0.0021 | 0.8621 | 0.7500 | 0.9674 | 0.6250 |
| 151 | 1107 | CAUUACUCCAGCUGACAAA | UUUGUCAGCUGGAGUAAUG | 0.0265 | 0.0224 | 0.6552 | 0.7500 | 1.0000 | 10.000 |
| 151 | 1152 | CUCCAUCACCGAUGUCACU | AGUGACAUCGGUGAUGGAG | 0.0469 | 0.0111 | 0.6034 | 0.5000 | 0.9076 | 0.8750 |
| 152 | 1172 | UCCUCAAAAGACACUUCCA | UGGAAGUGUCUUUUGAGGA | 0.0607 | 0.0286 | 0.5172 | 0.5000 | 1.0000 | 10.000 |
| 152 | 1040 | UGGUGGCAAGUGAUUAUGA | UCAUAAUCACUUGCCACCA | 0.1078 | 0.0021 | 0.6207 | 0.5000 | 1.0000 | 10.000 |
| 152 | 1384 | AGAUCACUUUACCUGCGUU | AACGCAGGUAAAGUGAUCU | 0.0264 | 0.0094 | 0.5776 | 0.7500 | 0.9402 | 0.7500 |
| 153 | 923 | CGACAAGCAUCAUCAACAC | GUGUUGAUGAUGCUUGUCG | 0.4184 | 0.0116 | 0.5000 | 0.7500 | 0.9674 | 0.6250 |
| 154 | 674 | CUGAUGUUGAUUGGCAAAG | CUUUGCCAAUCAACAUCAG | 0.0538 | 0.0020 | 0.6466 | 0.5000 | 1.0000 | 10.000 |
| 154 | 1134 | AGGUUUUGUUCUUGGUCAC | GUGACCAAGAACAAAACCU | 0.0636 | 0.0019 | 0.5517 | 0.5000 | 1.0000 | 10.000 |
| 155 | 63 | GAUGCGCAAAACCAAGCUU | AAGCUUGGUUUUGCGCAUC | 0.0330 | 0.0019 | 0.5431 | 0.7500 | 0.9076 | 0.7500 |
| 155 | 1081 | CCCCACUUUAAAUCUCUUG | CAAGAGAUUUAAAGUGGGG | 0.0239 | 0.0113 | 0.8448 | 0.7500 | 1.0000 | 10.000 |
| 156 | 1098 | UGGUCAAACCAUUACUCCA | UGGAGUAAUGGUUUGACCA | 0.6596 | 0.0374 | 0.5000 | 0.5000 | 0.9185 | 0.6250 |
| 157 | 710 | CCCAAUAUAGAAACGUGUG | CACACGUUUCUAUAUUGGG | 0.0224 | 0.0061 | 0.6983 | 0.7500 | 1.0000 | 10.000 |
| 157 | 993 | AGUUGAGCUGGAUACCUAC | GUAGGUAUCCAGCUCAACU | 0.1736 | 0.0019 | 0.5259 | 0.5000 | 0.9891 | 0.6250 |
| 158 | 1169 | CUUUCCUCAAAAGACACUU | AAGUGUCUUUUGAGGAAAG | 0.0484 | 0.0445 | 0.5000 | 0.7500 | 1.0000 | 10.000 |
| 158 | 667 | UGCAACCCUGAUGUUGAUU | AAUCAACAUCAGGGUUGCA | 0.0204 | 0.0120 | 0.8534 | 0.7500 | 0.9076 | 0.6250 |
| 159 | 799 | UUCUGCACGGAAUUUGGAU | AUCCAAAUUCCGUGCAGAA | 0.0339 | 0.0229 | 0.4914 | 0.5000 | 1.0000 | 10.000 |
| 161 | 521 | GCCCGAUGGAGAAAGUACG | CGUACUUUCUCCAUCGGGC | 0.1535 | 0.0016 | 0.7241 | 0.5000 | 1.0000 | 10.000 |
| 161 | 1139 | UUGUUCUUGGUCACUCCAU | AUGGAGUGACCAAGAACAA | 0.2457 | 0.0053 | 0.4914 | 0.5000 | 0.9185 | 0.6250 |
| 161 | 164 | GUGUUGUCCUCGAUGAAGU | ACUUCAUCGAGGACAACAC | 0.0198 | 0.0044 | 0.5862 | 0.5000 | 0.9457 | 0.6250 |
| 162 | 1050 | UGAUUAUGAUUUGGACUUU | AAAGUCCAAAUCAUAAUCA | 0.0194 | 0.0139 | 0.7328 | 0.7500 | 1.0000 | 10.000 |
| 162 | 675 | UGAUGUUGAUUGGCAAAGA | UCUUUGCCAAUCAACAUCA | 0.0474 | 0.0017 | 0.5086 | 0.5000 | 1.0000 | 10.000 |
| 162 | 1362 | CCUCUUUGAGAUACCAAGC | GCUUGGUAUCUCAAAGAGG | 0.0554 | 0.0021 | 0.4914 | 0.5000 | 0.9891 | 0.6250 |
| 163 | 1140 | UGUUCUUGGUCACUCCAUC | GAUGGAGUGACCAAGAACA | 0.2956 | 0.0055 | 0.5345 | 0.5000 | 0.9185 | 0.7500 |
| 163 | 676 | GAUGUUGAUUGGCAAAGAU | AUCUUUGCCAAUCAACAUC | 0.0424 | 0.0015 | 0.5000 | 0.7500 | 1.0000 | 10.000 |
| 163 | 1164 | UGUCACUUUCCUCAAAAGA | UCUUUUGAGGAAAGUGACA | 0.1839 | 0.0387 | 0.4914 | 0.5000 | 1.0000 | 10.000 |
| 163 | 1176 | CAAAAGACACUUCCACAUG | CAUGUGGAAGUGUCUUUUG | 0.0189 | 0.0069 | 0.4914 | 0.5000 | 1.0000 | 10.000 |
| 164 | 1150 | CACUCCAUCACCGAUGUCA | UGACAUCGGUGAUGGAGUG | 0.0430 | 0.0133 | 0.5603 | 0.5000 | 0.9076 | 0.8750 |
| 164 | 1245 | AGCCAUCCUCUCCUUUGCA | UGCAAAGGAGAGGAUGGCU | 0.3275 | 0.1013 | 0.4914 | 0.5000 | 1.0000 | 10.000 |
| 164 | 1033 | GACAUCGUGGUGGCAAGUG | CACUUGCCACCACGAUGUC | 0.1058 | 0.0014 | 0.5259 | 0.5000 | 1.0000 | 10.000 |
| 165 | 990 | GGGAGUUGAGCUGGAUACC | GGUAUCCAGCUCAACUCCC | 0.1897 | 0.0312 | 0.6379 | 0.5000 | 1.0000 | 10.000 |
| 165 | 1001 | UGGAUACCUACACCAUGAU | AUCAUGGUGUAGGUAUCCA | 0.0183 | 0.0057 | 0.6724 | 0.7500 | 0.9837 | 0.6250 |
| 166 | 584 | UUCUUUACACCAGGAUGAU | AUCAUCCUGGUGUAAAGAA | 0.0459 | 0.0092 | 0.4914 | 0.5000 | 0.9837 | 0.7500 |
| 166 | 170 | UCCUCGAUGAAGUCAUUUU | AAAAUGACUUCAUCGAGGA | 0.0186 | 0.0014 | 0.8707 | 0.7500 | 0.9457 | 0.6250 |
| 166 | 980 | GACACUAUGAGGGAGUUGA | UCAACUCCCUCAUAGUGUC | 0.0177 | 0.0016 | 0.5862 | 0.5000 | 1.0000 | 10.000 |
| 167 | 98 | ACGGUGUGUUCAACCCUGA | UCAGGGUUGAACACACCGU | 0.0497 | 0.0014 | 0.5431 | 0.5000 | 0.9239 | 0.7500 |
| 167 | 979 | AGACACUAUGAGGGAGUUG | CAACUCCCUCAUAGUGUCU | 0.0176 | 0.0014 | 0.5776 | 0.5000 | 1.0000 | 10.000 |
| 167 | 703 | CAUUUCGCCCAAUAUAGAA | UUCUAUAUUGGGCGAAAUG | 0.1891 | 0.0032 | 0.4828 | 0.7500 | 1.0000 | 10.000 |
| 168 | 633 | AAACAACGGACCGCAGAUU | AAUCUGCGGUCCGUUGUUU | 0.0200 | 0.0172 | 0.4914 | 0.5000 | 1.0000 | 10.000 |
| 168 | 1034 | ACAUCGUGGUGGCAAGUGA | UCACUUGCCACCACGAUGU | 0.2637 | 0.0019 | 0.4828 | 0.5000 | 1.0000 | 10.000 |
| 169 | 919 | UCCGCGACAAGCAUCAUCA | UGAUGAUGCUUGUCGCGGA | 0.0736 | 0.0030 | 0.6379 | 0.5000 | 0.9348 | 0.7500 |
| 169 | 833 | AGUGGAUUCUAAAGACUCU | AGAGUCUUUAGAAUCCACU | 0.0468 | 0.0018 | 0.4828 | 0.5000 | 0.9891 | 0.7500 |
| 169 | 621 | ACAAAUGCACUCAAACAAC | GUUGUUUGAGUGCAUUUGU | 0.1296 | 0.0013 | 0.5690 | 0.5000 | 0.9837 | 0.7500 |
| 169 | 854 | UGAACACAGAACACGCCUA | UAGGCGUGUUCUGUGUUCA | 0.0166 | 0.0044 | 0.5603 | 0.7500 | 1.0000 | 10.000 |
| 170 | 689 | AAAGAUUUGGCACCCAUUU | AAAUGGGUGCCAAAUCUUU | 0.0238 | 0.0013 | 0.5000 | 0.5000 | 0.9674 | 0.7500 |
| 170 | 1158 | CACCGAUGUCACUUUCCUC | GAGGAAAGUGACAUCGGUG | 0.4320 | 0.0160 | 0.4828 | 0.5000 | 1.0000 | 10.000 |
| 170 | 800 | UCUGCACGGAAUUUGGAUU | AAUCCAAAUUCCGUGCAGA | 0.0157 | 0.0139 | 0.7155 | 0.7500 | 1.0000 | 10.000 |
| 171 | 582 | CAUUCUUUACACCAGGAUG | CAUCCUGGUGUAAAGAAUG | 0.0383 | 0.0047 | 0.5000 | 0.5000 | 0.9891 | 0.6250 |
| 171 | 57 | CCACGUGAUGCGCAAAACC | GGUUUUGCGCAUCACGUGG | 0.0879 | 0.0013 | 0.5000 | 0.5000 | 0.9185 | 0.7500 |
| 171 | 876 | GAACAAACGCAUUGUGGUU | AACCACAAUGCGUUUGUUC | 0.0147 | 0.0086 | 0.5259 | 0.7500 | 0.9674 | 0.7500 |
| 172 | 1035 | CAUCGUGGUGGCAAGUGAU | AUCACUUGCCACCACGAUG | 0.2720 | 0.0032 | 0.4741 | 0.7500 | 1.0000 | 10.000 |
| 172 | 1002 | GGAUACCUACACCAUGAUC | GAUCAUGGUGUAGGUAUCC | 0.0146 | 0.0029 | 0.6897 | 0.7500 | 1.0000 | 10.000 |
| 173 | 609 | CAGAUUUUGUGCACAAAUG | CAUUUGUGCACAAAAUCUG | 0.0203 | 0.0039 | 0.6293 | 0.5000 | 0.9565 | 0.7500 |
| 173 | 1252 | CUCUCCUUUGCACGCCGUG | CACGGCGUGCAAAGGAGAG | 0.0133 | 0.0082 | 0.5172 | 0.5000 | 1.0000 | 10.000 |
| 173 | 879 | CAAACGCAUUGUGGUUGAA | UUCAACCACAAUGCGUUUG | 0.0168 | 0.0087 | 0.4741 | 0.7500 | 1.0000 | 10.000 |
| 173 | 1032 | CGACAUCGUGGUGGCAAGU | ACUUGCCACCACGAUGUCG | 0.0538 | 0.0012 | 0.5862 | 0.7500 | 1.0000 | 10.000 |
| 174 | 1167 | CACUUUCCUCAAAAGACAC | GUGUCUUUUGAGGAAAGUG | 0.1209 | 0.0826 | 0.5000 | 0.5000 | 1.0000 | 10.000 |
| 174 | 677 | AUGUUGAUUGGCAAAGAUU | AAUCUUUGCCAAUCAACAU | 0.0276 | 0.0012 | 0.6466 | 0.5000 | 1.0000 | 10.000 |
| 174 | 465 | CAUGGAGAACAGAGAAUAC | GUAUUCUCUGUUCUCCAUG | 0.0677 | 0.0061 | 0.4741 | 0.5000 | 1.0000 | 10.000 |
| 174 | 1051 | GAUUAUGAUUUGGACUUUG | CAAAGUCCAAAUCAUAAUC | 0.0129 | 0.0096 | 0.5345 | 0.5000 | 1.0000 | 10.000 |
| 175 | 599 | UGAUGAUAGGCAGAUUUUG | CAAAAUCUGCCUAUCAUCA | 0.0650 | 0.0016 | 0.5862 | 0.5000 | 1.0000 | 10.000 |
| 175 | 153 | GCUGAACGAAGGUGUUGUC | GACAACACCUUCGUUCAGC | 0.0247 | 0.0012 | 0.5345 | 0.7500 | 1.0000 | 10.000 |
| 175 | 632 | CAAACAACGGACCGCAGAU | AUCUGCGGUCCGUUGUUUG | 0.0305 | 0.0255 | 0.4655 | 0.7500 | 1.0000 | 10.000 |
| 175 | 1260 | UGCACGCCGUGGGACCAUA | UAUGGUCCCACGGCGUGCA | 0.0125 | 0.0012 | 0.8190 | 0.7500 | 0.9457 | 10.000 |
| 176 | 1045 | GCAAGUGAUUAUGAUUUGG | CCAAAUCAUAAUCACUUGC | 0.0430 | 0.0258 | 0.5086 | 0.5000 | 1.0000 | 10.000 |
| 176 | 1031 | ACGACAUCGUGGUGGCAAG | CUUGCCACCACGAUGUCGU | 0.0337 | 0.0011 | 0.6379 | 0.5000 | 0.9511 | 0.6250 |
| 176 | 666 | UUGCAACCCUGAUGUUGAU | AUCAACAUCAGGGUUGCAA | 0.0264 | 0.0138 | 0.4655 | 0.5000 | 0.9076 | 0.6250 |
| 176 | 1084 | CACUUUAAAUCUCUUGGUC | GACCAAGAGAUUUAAAGUG | 0.0121 | 0.0064 | 0.4741 | 0.5000 | 1.0000 | 10.000 |
| 177 | 942 | GAUUUUGAACAACAUCUAC | GUAGAUGUUGUUCAAAAUC | 0.3445 | 0.0773 | 0.5000 | 0.5000 | 1.0000 | 10.000 |
| 177 | 551 | CUCGCAUCGUCGAUGUCCU | AGGACAUCGACGAUGCGAG | 0.1162 | 0.0014 | 0.4655 | 0.5000 | 0.9293 | 0.6250 |
| 177 | 519 | UCGCCCGAUGGAGAAAGUA | UACUUUCUCCAUCGGGCGA | 0.1853 | 0.0010 | 0.6121 | 0.7500 | 0.9348 | 0.6250 |
| 177 | 1083 | CCACUUUAAAUCUCUUGGU | ACCAAGAGAUUUAAAGUGG | 0.0119 | 0.0095 | 0.5000 | 0.7500 | 1.0000 | 10.000 |

*^a^*The position of the first siRNA nt within the complementary 3D sequence is indicated. *^b^* Accessibility computed for a 6-8 nt window starting at the 3’ end of the target sequence (siRNA seed region). ^C^ Accessibility computed for a 12-16 nt window starting at the 3’ end of the target sequence. *^d,e^* Sequence(d) and energy (e)- based asymmetry criteria that specify strand selection as defined in RNAxs. *^f^* Minimal free folding-energy of the guide strand, as computed using RNAfold. *^g^* Number of paired nt among the first four nts at the 5’ end and 3’ end of the guide strand.
